# A Systematic Review of the Assessment of Support Needs in People with Intellectual and Developmental Disabilities

**DOI:** 10.3390/ijerph17249494

**Published:** 2020-12-18

**Authors:** Miguel A. Verdugo, Virginia Aguayo, Victor B. Arias, Laura García-Domínguez

**Affiliations:** Institute on Community Integration, University of Salamanca, 37005 Salamanca, Spain; verdugo@usal.es (M.A.V.); vbarias@usal.es (V.B.A.); lauragarciad@usal.es (L.G.-D.)

**Keywords:** support needs, assessment, quality of life, intervention, disability, systematic review

## Abstract

An evaluation of support needs is fundamental to the provision of services to people with intellectual and developmental disabilities. Services should be organized by considering the support that people need to improve their quality of life and enforce their rights as citizens. This systematic review is conducted to analyze the rigor and usefulness of the available standardized tools for assessing support needs, as well as the uses of their results. Several databases were consulted, including Web of Sciences, Scopus, PubMed, ProQuest Central, PsycInfo, ERIC, and CINAHL, and the 86 documents that met the review criteria were organized into four sections: (a) measurement tools, (b) descriptive/correlational studies, (c) predictive studies, and (d) interventions. The results showed that age, level of intellectual disability, adaptive behavior skills, the number and type of associated disabilities, and medical and behavioral needs affected the support needs of people with disabilities. Quality of life outcomes have been predicted by the individual’s support needs, explaining a significant percentage of their variability. The findings are useful in guiding assessments and planning interventions. Further research should address the effectiveness of specific support strategies and the development of social policies and indicators for inclusion that involve assessing support needs.

## 1. Introduction

The concept of “support needs” refers to the pattern and intensity of supports that are necessary for a person when it comes to participating in typical activities [1]. Understanding individuals based on their need for supports is the main premise of the support paradigm. By assuming this paradigm, organizations become the coordinators of support that people with disabilities and their families need, and which are often related to access to inclusive education, supported employment, or independent living, among other rights [1,2,3,4,5,6,7]. To the extent that the supports provided are aligned with the person’s needs and their objectives and desires, the person’s functioning in their environment will improve.

An individual’s support needs are used as the bases of developing individualized support plans, and aggregate data on the support needs of many individuals aim to improve organizational efficiency and resource allocation [8,9,10,11,12]. The support needs assessment serves to design individualized and generic support systems that enhance the quality of life for individuals with disabilities and their families, when maintained over time. Thus, the assessment of support needs is part of best practices in intellectual and developmental disabilities [13,14,15,16,17,18].

The challenge, therefore, has been to find a support needs assessment measure that captures all the influential variables in the planning of individual interventions, and which also contributes to resource allocation [19,20]. In fact, in one of the first publications on support needs assessment, Thompson et al. (2002) recognized that this is a “slippery construct” that requires specific procedures to systematically identify support needs in different activities in different contexts [21].

Over time, support needs have been assessed by using different methodologies, including clinical judgment, functional competency measures, estimation of educational and health needs, and standardized tools [14,19,21,22]. First, clinical judgment relies on expert opinion about the level of an individual’s support needs, usually considering different intensities (e.g., low, medium, and high). Second, scales of adaptive behavior and functional competency (e.g., daily living activities) have informed support needs by assuming that an individual’s decreased skills relate to increased support needs. Third, support needs have been understood as health care needs, in terms of “complex health needs,” “complex support needs,” or as educational support needs [23,24,25]. Their assessment considers the presence of different disabilities and focuses on specific areas (health, education). Finally, standardized scales have been developed to assess support needs. However, their validation has been hampered by the lack of criterion variables to contrast the measurements.

Although there is no agreement among researchers on the best support needs tool, it seems that a standardized and objective measure, as opposed to other forms of psychological assessment, could be a useful facilitator for planning teams within support provider systems [21,26,27,28]. Such an assessment should be responsive to changes over time, capture the medical, behavioral, and day-to-day needs of the person’s life in multiple settings, and serve the purposes of planning and resource allocation. The assessment should be integrated into a holistic support planification and implementation process that considers the person’s goals and embraces the person’s self-determination to decide on activities relevant to him or her [4,5,6].

In order to provide this measure, the American Association on Intellectual and Developmental Disabilities (AAIDD) elaborated the supports intensity scale (SIS). The SIS [28] aims to facilitate the implementation of the support model in service delivery organizations. It is intended to provide a standardized measure of the intensity of support that a person with an intellectual or developmental disability requires in order to perform daily activities.

The SIS has been translated and adapted to more than 16 countries, becoming a reference tool for measuring support needs. However, it has not been free of criticism [19,29,30]. The debate mainly focused on the adequacy or otherwise of estimating support needs through a standardized set of activities that may not be part of a person’s life. In addition, its application procedures have been discussed, as well as the validation of its measurement model and its generalization among groups of individuals. Recent research also identified a ceiling effect on the scale and warns about the difficulty of its use in people with greater support needs [31,32,33].

### The Present Study

The purpose of this study is to critically examine the state of support needs assessment for people with intellectual and developmental disabilities. The overall objective is to analyze the rigor and usefulness of the available standardized tools for assessing support needs, as well as the uses of their results. The specific objectives are as follows: (1) to study the standardized support needs tools and their psychometric evidence; (2) to examine the relationship between support needs and other variables that have an influence on them; (3) to describe the impact of support needs on desired outcomes and resource allocation; and (4) to consider implemented interventions that use levels of support needs to evaluate their effectiveness.

## 2. Materials and Methods

A systematic review was conducted on the measurement of support needs in people with disabilities. The review followed a protocol based on the recommendations of the Cochrane Collaboration and the PRISMA statements [34,35,36].

### 2.1. Search Strategy

Studies were identified by searching electronic databases, scanning the references of full-text documents, and consulting with experts in the field. No restrictions on language, date, or publication status were imposed. The search was conducted on PubMed (1992–present), Scopus (1978–present), Web of Science (using the WOS Core Collection, Current Contents Connect, MEDLINE, and SciELO databases; 1992–present), PsycInfo (1993–present), ERIC (1988–present), CINAHL Complete (1990–present), CSIC (2003–present), and ProQuest Central (1990–present). The first search was carried out in June 2018 and was updated in March 2020. The last search was conducted independently by two members of the research team in September 2020. The recovered documents were handled with the bibliographic manager, Mendeley Desktop v 1.19.4 (2008, Glyph & Cog, LLC, Petaluma, CA, USA), through which the duplicate documents were automatically eliminated.

The databases were queried using titles, abstracts, and the keywords “support needs” and “disability”. In addition, the terms “measurement”, “assessment” and “evaluation” were included. When available, the corresponding Medical Subject Headings (MeSH) terms were used. Participants of any age were considered, and the search was not limited to a specific type of disability. The complete search strategy is included in Appendix A.

### 2.2. Selection Criteria

The documents’ selection was carried out independently by two researchers, and disagreements between them were settled by consensus. First, previously unidentified duplicates were eliminated, and so were conference proceedings, editorials, letters, book reviews, and press releases. Then, the titles and abstracts of the remaining articles were screened to identify eligible papers reviewed in full text.

To be included in the review, the document must report on persons with disabilities’ support needs through a standardized assessment. The procedure for data collection and analysis must also be described. The disabilities admitted are developmental disabilities, including intellectual disabilities and related syndromes (e.g., Down syndrome), autism spectrum disorder and/or cerebral palsy, and psychiatric, motor, and sensory disabilities. The exclusion criteria are (a) assessment of the support needs of persons without disabilities (i.e., temporary diseases, or caregiver/family support needs); (b) studies that do not evaluate support needs (i.e., measurements of adaptive behavior or functional competency, including employment, social and independent living skills); (c) theoretical reviews of the concept or measure of support needs that do not provide assessment data; (d) studies that do not use standardized tools for measuring support needs (e.g., qualitative studies, general estimates of levels of needs, and ad hoc questionnaires); and (e) standardized assessments that only consider a specific support domain (i.e., learning, employment, or health needs).

**Figure 1:**
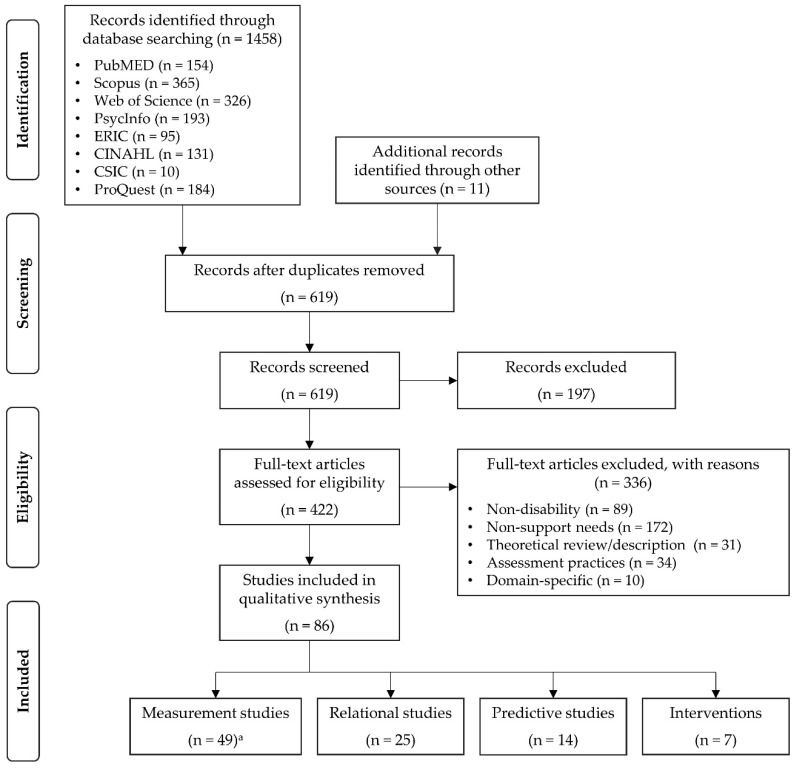
PRISMA flow diagram. ^a^ The measurement studies overlapped in 9 cases with the relational (n = 8) and predictive (n = 1) studies.

The documents resulting from the application of the inclusion and exclusion criteria are part of this review. Figure 1 shows a graph with the complete process of the selection of eligible sources.

### 2.3. Data Extraction

The selected studies were further examined. A summary sheet was developed following Cochrane recommendations for data extraction [36]. Details of the participants (n, age, setting, and diagnosis), the interventions, the results, and the study characteristics were included. The studies were classified into four categories based on the nature of their methodologies: validation of support needs instruments, descriptive-correlational studies, multivariate studies, and interventions.

(1) The measurement studies were grouped according to the scale used. The data extracted referred to the administration format, target group (age range and diagnosis), purpose and focus, addressed domains, scoring, and pieces of evidence of reliability and validity.
(2) In relational studies, support needs were correlated with variables of interest. The data collected related to the description of the variables and association indexes, if available.
(3) In multivariate studies, the results of support needs were a predictive variable in models of different complexity. Data collection focused on the results obtained, the variables included in the models, and the support needs’ effect on the target outcome.
(4) Finally, support needs acted as a factor of change in the intervention studies. In these cases, the components of the intervention, time, selection procedures, and outcomes were collected.

The data extraction protocol was developed a priori and agreed upon by the research team. The data extraction was performed by an author and verified by an independent qualifier to determine its accuracy. In case of disagreement on the extracted data, the articles were re-examined until a consensus was reached. Cochrane recommendations were followed to reduce the risk of bias in data extraction, and overlapping information from different articles was grouped into one work.

## 3. Results

A total of 1469 records were identified through electronic and second-hand searches and reduced to 619 after removing duplicate documents through the bibliographic manager. A posterior scanning allowed the elimination of another 106 duplicates plus 91 records from grey literature (which only provide a study’s abstract). The remaining 422 documents were reviewed in full text. The exclusion criteria application allowed the elimination of 336 records, keeping a total of 86 articles that met the predetermined criteria for inclusion and were further evaluated for review (see Figure 1).

Table 1 contains the characteristics of the studies included in the review, along with the details of their participants. As shown, the included studies involved samples of participants between the ages of 5 and 89, with intellectual and developmental disability being the most studied condition. Most of the studies were conducted in the United States, Australia, and Spain. Most of the selected articles aimed at validating instruments and describing variables that influence support needs. The SIS support intensity scales were the most frequent support needs assessment tools.

The results were divided into four categories: measurement tools, relational studies, predictive studies, and interventions.

### 3.1. Measurement Tools (n = 49)

Nine measures of support needs were found in the studies that met the selection criteria. Their characteristics are summarized in Table 2. The instruments were as follows: (a) Camberwell assessment of need for adults with developmental and intellectual disabilities (CANDID); (b) care and needs scale (CANS) and pediatric care and needs scale (PCANS); (c) supports intensity scale (SIS, SIS-A) and supports intensity scale—children’s version (SIS-C); (d) instrument for classification and assessment of support needs (I-CAN); (e) North Carolina—support needs assessment profile (NC-SNAP); (f) service need assessment profile (SNAP); and (g) support needs questionnaire (SNQ).

According to Table 2, seven out of nine instruments are for adults with intellectual and developmental disabilities, and four out of nine use the semi-structured interview. They all have in common the observation of the person with a disability in different aspects of daily life, including among others, health issues (either generic care or physical, mental and emotional health), behavioral challenges, personal care and domestic life activities. However, only SIS and I-CAN have been used for individualized support planning [8,10,20] and resource allocation [9,41], as compared to NC-SNAP and SNAP—used for funding purposes—and CANS, PCANS, CANDID and SNQ—used for support planning for persons with brain injury or those who use mental health services.

In terms of scoring, all measures involve different estimates of support needs through Likert scales of three, five, seven, or eight response options. All include estimates of the intensity of support needed, and four of them specify the frequency and/or estimated time of support needed to complete daily life activities. The support intensity varies from no support to full support, and only I-CAN makes a qualitative estimate of support needs. Solely CANDID includes the concept of “problem” as parallel to “need”.

The content validity of the measurement tools was based on expert opinion, a bibliographic search of relevant indicators, and pilot studies. Only SIS and SIS-C have used Rasch modeling [33,52] or factorial analysis to explore construct validity. The criterion measures are often adaptive behavior or competency scales. The evidence of internal consistency meets the standards, as are test-retest and interrater rates when reported.

The SIS support intensity scales collect the most psychometric evidence. The SIS scales were originally developed and validated using the classical test theory [94,96]. In these analyses, all the subsequent validations and adaptations were made. In this sense, the best fit model for measuring support needs comprised seven support dimensions and three method factors (see Appendix B). However, some studies warn about this measurement model’s utility due to (a) the excessive influence of the time factor and (b) the difficulty in discriminating scores at high levels of support needs [31,32,33,38,104].

### 3.2. Relational Studies (n = 25)

Studies highlight some variables related to the levels of required support. These variables are age, levels of intellectual disability and adaptive behavior, and the presence of different disabilities and health and behavioral needs.

#### 3.2.1. Age

The influence of age on support needs has been researched in at least 13 studies, with three of them having it as the primary focus [49,82,103]. Support needs decrease as the child grows [82], with more significant differences between the 5–10 and 11–16 age cohorts [33,49,97,102,103]. However, during the transition stage to adult life, between 16 and 22 years of age, support needs are again higher and present great variability, suggesting the need to evaluate supports and make effective plans in this period [79,81]. Although age does not appear to be related to support needs in adults [63,64,99,101], the presence of associated health conditions could influence this relationship [47,48].

#### 3.2.2. Levels of Intellectual Disability and Adaptive Behavior

The presence of significant limitations in intellectual functioning and adaptive behavior skills, both considered criteria for a diagnosis of intellectual disability, has been directly related to increased support needs in 22 studies. The traditional levels of intellectual functioning, mild (IQ 50–69), moderate (IQ 35–49), severe (IQ 20–34), and profound (IQ < 20), have been associated with scores in support needs, with positive and moderate-to-high strength relationships being obtained [55,65,84]. Kuppens et al. (2010) analyzed that relationship further. They conducted a measurement invariance study to test whether the model for measuring support needs was equivalent across different levels of intellectual disability. These authors could not establish invariance at the level of factor loads (i.e., weak invariance), suggesting that different groups do not respond in the same way to the measure of support needs [63].

Adaptive behavior has been negatively related to support needs (ranging from −0.18 [65] to −0.90 [57]), so that higher adaptive behavior skills have been associated with lower support needs [9,10,19,42,43,44,45,55,57,59,65,67,71,80,84,86,87,89,94,101]. Adaptive behavior measures have been used as a criterion measure of support needs instruments and, under the assumption that a reduction in the person’s skills is related to increased support needs, different scales of adaptive behavior and functional competence have been frequently used to report support needs.

#### 3.2.3. Presence of Multiple Needs and Health Conditions

The effect of multiple needs and health conditions on support needs has been analyzed by considering three aspects: the presence of additional medical and behavioral needs, the number of disabilities present, and the types of co-occurring disabilities.

First, the presence of medical and behavioral needs has been positively associated with support needs [38,58,65,71,79]. At higher levels of support needs, medical needs appear to have the greatest influence [38,79]. Medical needs are related to the presence of motor disabilities [40,87], although, in people with brain injury, the study of challenging behaviors has been frequent [61,72]. Behavioral needs have been associated with psychiatric [39,70,72] and autism spectrum disorders [84].

Second, the presence of additional conditions has been associated with a considerable need for supports [41,55,58,63,87], making it difficult to discriminate between support needs when more than three disabilities are present [55]. Kuppens et al. (2010) conducted an invariance study between two groups: people with one disability and people with more than one disability [63]. They found that, although both groups are equivalent in measuring support needs (i.e., they achieve strong invariance), the mean scores differ significantly between the two groups.

Finally, the studies have collected samples of participants with different disabilities to study their support needs. Specifically, the support needs of adults with psychiatric disorders [39,46,47,60,75,90], motor disability [40,61,72,87,89,91,92,93] or several of these [42,109]; and youth with autism spectrum disorders [83,84] and motor disability, primarily cerebral palsy [31,32,50,88] have been studied.

In adults, the greatest frequency of support needs relates to areas of employment and lifelong activities [109], as well as home, community and health [39,40,42,109]. In general, it appears that the scales may evaluate support needs in people with different disability conditions, although studies warn about a greater presence of disruptive behaviors in psychiatric disorders and of medical needs in motor disabilities. When they appear together with intellectual disability, the intensity of support needs is anyway greater [40,109].

Research on support needs in children with different disabilities has been less common than in adults. It appears that limitations in gross and fine motor function are associated with increased support needs [31,32,50], even when the different levels of intellectual disability are controlled [32]. As in the case of adults, youth with more severe motor disabilities had a greater quantity of medical support needs and fewer behavioral support needs. In the case of children with autism spectrum disorder [83,84], the greater needs for support are connected with social activities and behavioral needs.

### 3.3. Predictive Studies (n = 14)

Support needs have been used in multivariate studies, within models that predict the following types of outcomes: quality of life, self-determination, and support levels.

#### 3.3.1. Quality of Life and Self-Determination

Three studies have studied the impact of support needs on quality of life outcomes through regression analysis [8,20,86]. Lombardi et al. (2016) analyzed a model in which client characteristics, desires and goals, support needs, support strategies, and environmental factors combined to predict quality of life outcomes. They found that support needs explained 26.7% of the variability in quality of life outcomes, considering all eight domains [8]. Claes et al. (2012) include support needs as part of client characteristics and estimate that they affect 44.4% of quality of life outcomes, along with age, gender, mobility, and levels of intellectual disability [20]. Finally, Simões et al. (2016) found that support needs predicted 12% (when self-reporting) or 9% (when report-of-others) of quality of life outcomes, although the percentage varied by dimension (greater variance explained by the personal development domain and non-significant scores in the emotional well-being and material well-being domains). Moreover, they found that measures of adaptive behavior were stronger predictors compared to support needs on quality of life outcomes [86].

On the other hand, Vicente et al. (2015) studied the elements that influence the self-determination of students with disabilities, including personal factors (gender, age, intellectual disability level, and support needs) and school context variables (school setting, type of classroom placement, and experience with transition programs). The regression analysis shows that the support needs explained 15.9% of the variation in self-determination outcomes, where lower support needs were associated with higher self-determination [107].

#### 3.3.2. Resource Allocation

Better financing for people with disabilities and their families can be associated with the allocation of resources based on the general characteristics of needs. In this way, support needs scores can be arranged into levels, according to cut-off points, where each level is assigned specific resources in both individual and service budgets. In our review, we have found 10 studies that use support needs for this purpose of allocating resources or studying the best way to finance services for people with disabilities [9,10,11,12,43,44,59,90,98,110].

In general, there is a high compatibility between the levels of support needs after the application of a support needs measure and levels of support estimated by experts without using a standardized tool [59,90,98,110]. Moreover, several studies conclude that a measure of support needs is more predictive of resource allocation than general levels of need (e.g., limited, regular, extensive, or generalized care) estimated by adaptive behavior scales or functional competence measures [9,10,11,43,44]. Support needs were predictive of medical expenses in one study [12], with positive direction in that association (the higher the support needs, the higher the medical expenses).

### 3.4. Interventions (n = 7)

The support needs scores have been used to assess the effectiveness of six interventions [51,62,68,73,74,92,108], focused on the development of life skills or related to employment. Of these, only Tate et al. (2019) found no effect on support needs, as assessed through CANS, after applying a coaching program for transition to adulthood to youths with brain injury [92].

Regarding skills development, Koristas et al. (2008) assessed the effect of active support training of 12 adults with intellectual disability, with the reduction of perceived support needs being one of the indicators of effectiveness, which was attributed to the increased opportunity to develop skills [62]. Prohn et al. (2018) concluded that their post-secondary programs (focused on skills development and independent living) influenced the adaptive behavior skills and support needs of six students with intellectual disabilities [68]. Sanjo et al. (2018, 2019) studied the effects of self-management training for people with intellectual disabilities through skills training for their participants and their caregivers, resulting in a significant decrease of their support needs at 9 and 12 months [73,74].

With reference to employment, Gomes-Machado et al. (2016) assessed the effects of a vocational training program on the adaptive behavior and employability skills of 43 youths with intellectual disabilities, obtaining a 50% reduction of support needs in various areas [51]. Finally, Wehman et al. (2016) evaluated an employment program for 49 youths with intellectual disability and autism, obtaining meaningful reductions in support needs over the control group after 3 and 12 months of intervention [108].

## 4. Discussion

### 4.1. Present Study

This systematic review aims to investigate the assessment of the support needs of people with disabilities. For this purpose, 86 studies were analyzed. Firstly, nine standardized assessment scales account for support needs, capturing the intensity of support in different daily living areas. Secondly, higher support needs have been related to growing in age (in childhood), higher levels of intellectual disability, and the presence of other health and behavioral conditions. Thirdly, support needs have been used to predict outcomes in quality of life, self-determination, and resource allocation, indicating significant associations. Lastly, some interventions showed that support needs could be considered a factor of change in assessing a program’s effectiveness. These results are broader explained below.

First, the support needs of people with disabilities have been analyzed using nine instruments: CANDID for people with psychiatric disorders [112]; CANS [89,91] and PCANS [88] for people with brain injuries; and NC-SNAP [59], SIS [39,40,42,45,46,60,63,64,66,77,78,80,81,85,87,94,95,99,100,101], SIS-C [31,32,33,37,52,53,56,76,77,78,82,83,96,97,104,105,106], SNQ [48], I-CAN [19,41,69,70,71], and SNAP [54,55] for people with intellectual and developmental disabilities mostly, but also their validation samples include psychiatric disorders, autism spectrum disorders, and physical disabilities. The SIS-C and PCANS scales are the only ones focused on children.

Validation work on the scales has included samples from 40 to 130,000 participants. It seems that I-CAN and SIS scales are the most researched measures of support needs. The main difference between them is that the I-CAN assumes the World Health Organization framework [113], while the SIS assumes the AAIDD functioning model [4]. The I-CAN is answered by considering the person’s wishes in a relevant context, while the SIS provides standardized activities. The SIS’s main advantage is its widespread use in more than 15 countries, while the I-CAN is only used in Australia and Singapore [114].

Second, studies have identified certain variables that generally influence the support needs profiles of people with disabilities. In this review, we have analyzed age, levels of intellectual disability, adaptive behavior skills, and other health conditions, as these are the most recurrent in the research. The relationship between support needs and these variables is relevant to guide planning and service allocation [8,9,10,20]. Thus, knowing that support needs are significantly higher during the transition to adulthood (the ages of 16–21) may motivate the implementation of interventions at this stage [68,82,115]. Furthermore, studies that relate support needs to age recommend reassessing at different educational stages for better support planning, developing vocational and training programs, and ensuring supports for independent living [116,117,118].

The relationship between support needs and levels of intellectual disability demands further attention. While it seems that higher levels of intellectual disability are related to higher levels of support needs, the methodology used to reach that conclusion should be examined. First, it is problematic to obtain a valid and reliable measure of intellectual functioning with IQs below 50 [119,120], which has led most studies to make an overall estimate of intellectual functioning. Second, these levels have been related with a continuous score of support needs, obtaining significant correlations in indices that dismiss the data’s ordinal nature. When the dispersion of support needs scores has been explored [32,63], overlaps between the different groups have been found, indicating that they are not significantly different despite their means. The accumulation of scores in the higher part of the variable also denotes a ceiling effect of support needs in people with more severe intellectual disability levels, which may demand further research [31,32,33,38].

The relationship between lower support needs and higher adaptive behavior skills has led some authors to discuss whether this is one or two different constructs [43,45,55,57,65,86]. While a measure of adaptive behavior reflects the level of competence in behavioral, social, and/or practical skills—as defined by the AAIDD definition of intellectual disability [4], a measure of support needs is indicative of the intensity of support needed—in type, frequency, and time—to complete an activity [57,67,86,121,122]. Practitioners and organizations should take into account the differences between both measurements when generalizing support needs results and that the adaptive behavior scores do not provide explicit information about the level of support needs (which captures more elements for planning a support system).

Support needs measures have been provided for individuals with intellectual and developmental disabilities, physical disabilities (mainly due to brain injury and cerebral palsy), psychiatric disorders, and autism spectrum disorders. The greatest need for support seems to be related to greater health problems. However, the relationship may not be predictive (i.e., support needs predict the diagnosis), but rather, it may be the impact of other variables (e.g., restrictions in health services or delayed acquisition of skills) that best explains the impact of the additional disabilities on support needs.

Third, support needs have been considered as elements in multivariate models to predict outcomes in quality of life, self-determination and resource allocation. However, these studies are still scarce. Lombardi et al. (2016) found that support needs explained 27% of the variability in the quality of life outcomes [8]. This percentage is relevant if we consider that support needs rely on the person-environment relationship, so therefore, some level of support might enhance an individual’s quality of life. Further research is crucial to align support provision with valued outcomes, such as quality of life and self-determination [13,123,124,125].

Finally, four studies examined support needs as factors of change. These studies are preliminary and do not provide enough evidence of effectiveness. However, they suggest that adopting an organizational management system focused on the person and on support coordination could be more effective than other service provision forms. Lists of common professional interventions and support strategies have often been proposed [126], and it is a current challenge to test them through quality methodology studies.

### 4.2. Limitations

Two additional exclusion criteria would have been desirable in this review. The first is a criterion based on the methodological quality of the selected studies. The advantages of including this criterion rely on eliminating studies with small samples or lacking quality. However, this review aimed to report on the different support needs’ measures, including their generalization, so the inclusion of such a criterion would have prevented a broad perspective. Nevertheless, we encourage researchers to apply a more exhaustive analysis of the present review results.

The second criterion relates to the inclusion of different types of disabilities. Although this review focuses on people with intellectual and developmental disabilities, we have omitted this selection criterion because the assessment of support needs is extended to other populations using the same scales in many cases.

Other limitation of this review refers to the generalization of the results. The methodological variability among studies has not allowed us to contrast their results through association indices. Most studies offered indicators that depend on the size of the sample and do not consider the influence of different variables (e.g., levels of intellectual disability), that we argue affects the evaluation of support needs. Therefore, we have presented an aggregate data of evidence without further analysis.

### 4.3. Future Research

The extensive literature suggests that some variables are closely related to support needs: age, type of disability, additional needs, and levels of intellectual disability and adaptive behavior. However, very few studies analyzed support needs profiles considering different support domains and specific characteristics. On the contrary, the studies aggregate data on the support needs. Further research is required to add to the description of the profiles of people with disabilities involved in assessing their support needs—especially, aspects related to the environment in which they live, and the accessibility of community service.

Although support needs profiles are individualized, more comprehensive profile descriptions could guide research on particular support strategies. In this regard, very few studies have been published on the effectiveness of different interventions or support strategies. These studies should relate support needs to desired personal outcomes (e.g., quality of life) or organizational effectiveness and efficiency.

In relation to scales of assessment, forthcoming research is needed on the measurement of support needs of people with greater levels of intellectual disability, as well as on the generalization of support needs to different conditions.

## 5. Conclusions

The assessment of support needs has focused mostly on the adult population with developmental disabilities, although its application extends to children and other conditions. Most research has focused on the development and validation of assessment scales, with few studies investigating the effectiveness of using support needs to achieve desired personal outcomes and improve organizational effectiveness and efficiency. There is a need for future research to examine support needs in people with higher levels of need and further analyze the methodological quality of the scales used in the support needs assessment.

## Figures and Tables

**Table 1:** Studies Comprising the Systematic Review (n = 86).

Study	Country	N	Age	Condition ^a^	Measure ^b^	Study Category
Adam-Alcocer & Giné (2013) [37]	Spain	33	5–15	ID	SIS-C	Measurement
Aguayo, Arias, et al. (2019) [31]	Spain	210	5–16	ID, MD	SIS-C	Measurement, Relational
Aguayo, Verdugo, et al. (2019) [32]	Spain	713 + 286	5–16	ID, MD	SIS-C	Measurement, Relational
Arias, Aguayo, et al. (2020) [38]	Spain	911	5–16	IDD	SIS-C	Relational
Arnkelsson & Sigurdsson (2014) [39]	Iceland	121	21–74	PD	SIS	Measurement
Arnkelsson & Sigurdsson (2016) [40]	Iceland	207	18–79	ID + MD	SIS	Measurement, Relational
Arnold et al. (2009) [19]	Australia	1012	-	IDD	I-CAN	Measurement
Arnold et al. (2014) [10]	Australia	163	15–55	MD, ID, SD, ABI	I-CAN-Brief Research	Predictive
Arnold et al. (2015) [41]	Australia	186 + 41	24–65	MD, ID, SD, ABI	I-CAN	Measurement
Bossaert et al. (2009) [42]	Belgium	1303	20–86	MD, ID, SD, ABI, ASD	SIS	Measurement
Brown et al. (2009) [43]	Canada	40	18–45	ID	SIS	Predictive
Chou et al. (2013) [44]	Taiwan	139	16–53	ID	SIS	Predictive
Claes et al. (2009) [45]	The Netherlands	29 + 75	14–81	IDD	SIS	Measurement
Claes et al. (2012) [20]	The Netherlands	186	19–83	IDD	SIS	Predictive
Cruz et al. (2010) [46]	Mexico	85	-	PD	SIS	Measurement
Cruz et al. (2013) [47]	Mexico	182	-	PD	SIS	Relational
Davis et al. (2015) [48]	United Kingdom	82	24–76	PD	SNQ	Measurement
Dinora et al. (2020) [12]	US	522	18–64	IDD, PD, MD, ASD	SIS	Predictive
Giné et al. (2014) [9]	Spain	134	-	ID	SIS	Predictive
Giné, Adam, et al. (2017) [49]	Spain	949	5–16	ID	SIS-C	Relational
Golubović et al. (2020) [50]	Serbia	40	7–14	ID, MD	SIS-C	Relational
Gomes-Machado et al. (2016) [51]	Brazil	43	18–28	ID	SIS	Intervention
Guillén et al. (2015) [52]	Spain	143	5–16	ID	SIS-C	Measurement
Guillén et al. (2017) [53]	Spain	814 + 949	5–16	ID	SIS-C	Measurement
Guscia et al. (2005) [54]	Australia	318	19–73	ID, ABI, PD	SNAP	Measurement
Guscia et al. (2006) [55]	Australia	114	20–72	ID, MD	SNAP, SIS	Measurement, Relational
Hagiwara et al. (2019) [56]	US	3436	5–16	IDD	SIS-C	Measurement
Harries et al. (2005) [57]	Australia	80	20–72	ID	SIS	Relational
Harries et al. (2009) [58]	Australia	83	20–72	ID, MD, SD, ABI, ASD	SNAP, SIS	Relational
Hennike et al. (2006) [59]	US	553	-	IDD	NC-SNAP	Measurement, Predictive
Jenaro et al. (2011) [60]	Mexico	182	16–87	PD	SIS	Measurement
Kelly et al. (2008) [61]	Australia	190	18–65	ABI, BD	CANS	Relational
Koritsas et al. (2008) [62]	Australia	12	27–57	ID	SIS	Intervention
Kuppens et al. (2010) [63]	Belgium	14,862	20–89	IDD	SIS	Measurement, Relational
Lamoureux-Hébert & Morin (2009) [64]	Canada	245	16–75	ID	SIS	Measurement
Lamoureux-Hébert et al. (2010) [65]	Canada	191	16–75	ID	SIS	Relational
Lombardi et al. (2016) [8]	Italy	1285	16–80	IDD	SIS	Predictive
Morin & Cobigo (2009) [66]	Canada	42	16–68	ID	SIS	Measurement
Obremski (2014) [67]	US	102	5–16	IDD	SIS-C	Relational
Prohn et al. (2018) [68]	US	6	19–23	ID	SIS	Intervention
Riches (2003) [69]	Australia	116	13–50	IDD, ID, MD, PD	I-CAN	Measurement
Riches et al. (2009a, 2009b) [70,71]	Australia	1012	17–77	IDD, ID, MD, PD	I-CAN	Measurement, Relational
Sabaz et al. (2014) [72]	Australia	507	18–65	ABI	CANS	Relational
Sandjojo et al. (2018, 2019) [73,74]	The Netherlands	17	>18	ID	SIS	Relational
Schützwohl et al. (2016) [75]	Germany	371	18-65	ID, PD	CANDID	Relational
Seo, Shogren, et al. (2016) [76]	US+CA	129,864 + 4015	5–64	IDD	SIS, SIS-C	Measurement
Seo et al. (2016, 2017) [77,78]	US+CA	142	15–21	IDD	SIS, SIS-C	Measurement
Seo, Shogren, et al. (2017) [79]	US+CA	13,968	16–22	IDD	SIS	Predictive
Shogren et al. (2014, 2016) [80,81]	US+CA	139,129	16-80	IDD	SIS	Measurement
Shogren et al. (2015) [82]	US	4015	5–16	IDD	SIS-C	Relational
Shogren, Shaw, et al. (2017) [83]	US	2124	5–16	IDD, ASD	SIS-C	Measurement
Shogren, Wehmeyer, et al. (2017) [84]	US	2124 + 1861	5–16	IDD, ASD	SIS-C	Measurement, Relational
Shogren et al. (2018) [85]	US	82	21–79	IDD	SIS	Measurement
Simoes et al. (2016) [86]	Portugal	146	18–68	ID	SIS	Predictive
Smit et al. (2011) [87]	Belgium	65	21–60	MD	SIS	Measurement
Soo et al. (2008) [88]	Australia	32	5–17	ABI	PCANS	Measurement
Soo et al. (2010) [89]	Australia	68	16–70	ABI	CANS	Measurement
Tassé & Wehmeyer (2010) [90]	US	172 + 143	19–83	ID, PD	SIS	Predictive
Tate et al. (2004) [91]	Australia	67	-	ABI	CANS	Measurement
Tate et al. (2019) [92]	Australia	43	14–19	ABI	CANS	Intervention
Tate et al. (2020) [93]	Australia	131	-	ABI	CANS	Relational
Thompson et al. (2002) [94]	US	46	93	ID	SIS	Measurement
Thompson et al. (2008) [95]	US	40	20–69	ID	SIS	Measurement
Thompson et al. (2014) [96]	US	4015	5–16	IDD	SIS-C	Measurement
Thompson et al. (2020) [97]	Iceland	649 + 4015	5–16	IDD	SIS-C	Measurement
Tremblay & Morin (2015) [98]	Canada	30	18–56	ID	SIS	Predictive
Vega Córdoba et al. (2012, 2014) [99,100]	Chile	285	18–51	IDD	SIS	Measurement
Verdugo et al. (2010) [101]	Spain	885	15–76	ID	SIS	Measurement
Verdugo, Amor, et al. (2019) [102]	Spain	814 + 222	5–16	ID, no D	SIS-C	Measurement
Verdugo, Arias, et al. (2016) [103]	Spain	450	5–16	ID	SIS-C	Relational
Verdugo et al. (2016, 2019, 2020) [33,104,105]	Spain	814	5–16	ID	SIS-C	Measurement
Vicente et al. (2015) [106]	Spain	99	11-19	ID	SIS-C	Measurement
Vicente et al. (2019) [107]	Spain	232	11–19	ID	SIS-C	Predictive
Wehman et al. (2016) [108]	US	49	18–21	ASD	SIS	Intervention
Wehmeyer et al. (2009) [11]	US	274	19–83	IDD	SIS	Predictive
Wehmeyer et al. (2012) [109]	US	274	19–84	IDD, MD, PD, ASD	SIS	Relational
Weiss et al. (2009) [110]	Canada	50	-	ID	SIS	Predictive
Winkler et al. (2010) [111]	Australia	189	-	ABI	CANS	Relational
Xenitidis et al. (2000) [112]	United Kingdom	40	20–67	PD	CANDID	Measurement

^a^ ID = intellectual disability; IDD = intellectual and developmental disabilities; MD = motor disability; PD = psychiatric disorder; SD = sensorial disability; ABI = acquired brain injury, ASD = autism spectrum disorders. ^b^ CANDID = Camberwell assessment of need for adults with developmental and intellectual disabilities; CANS = care and needs scale; I-CAN = instrument for classification and assessment of support needs; NC-SNAP = North Carolina support needs assessment profile; PCANS = paediatric care and needs scale; SIS = supports intensity scale; SIS-C = supports intensity scale—children’s version; SNAP = service need assessment profile; SNQ = support needs questionnaire.

**Table 2:** Measurement Tools of Support Needs.

Measure ^a^	Administration Format	Age Range	Target Group	Purpose and Focus	Domains	Scoring	Validity and Reliability
CANDID [112]	Semi-structured interview to service users, their informal carers and staff	>16	People using community mental health services	It was developed by modifying the Camberwell assessment of need to make its content relevant to adults with learning disabilities and mental health problems. It aims to assess the needs of people who may require community care	Twenty-five domains, including basic needs (accommodation, food, public transportation, money and benefits); self-care/functional needs (self-care and daytime activities); health/safety needs (physical health, psychological distress, psychotic symptoms, safety); and social needs (company, intimate relationships and sexual expression)	Three-levels: no need/no serious problem, met need/moderate problem due to help given, and unmet need/serious problem	Test-retest: ICC = 0.69 to 0.86 Interrater: ICC = 0.93–0.97 Criterion validity: GAF
CANS [89,91]	Clinician reported questionnaire	>16	Brain injury	It assesses the support needs for everyday activities and community living of people with brain injury	Four domains: special needs, basic activities of daily living, instrumental activities of daily living, and informational and emotional supports	Section 1: items are endorsed if a support need is present. Section 2: from 0 = does not need contact to 7 = cannot be left alone	Test-retest: ICC = 0.98 Interrater: ICC = 0.93–0.96 Concurrent validity with Craig handicap and assessment reporting technique
I-CAN [19,41,69,70,71]	Semi-structured interview to service users, their informal carers and staff	>16	Developmental, genetic or acquired disabilities	It proposes a system to identifying and classifying support needs of people with disabilities based on the conceptual framework of the International Classification of Functioning, Disability and Health	Health and wellbeing: physical health, mental and emotional health, behavior of concern, health and support services; and activities and participation: applying knowledge, general tasks and demands, communication, self-care and domestic life, mobility, interpersonal interactions, life-long learning, and community social and civic life	Qualitative fields (‘I can… Goals… My Support Needs’), and quantitative fields scoring 0–5-point Likert scales regarding frequency and level of support	Cronbach alpha: 0.83 to 0.93 Internal consistency: ICC = 0.91–0.98 Test-retest: ICC = 0.94 Interrater: ICC = 0.96 Criterion validity: daytime support (0.40), 24 h support (0.27) and ICAP
NC-SNAP [59]	Stuff reported questionnaire	>16	Diverse disabilities	It measures the level or intensity of a person’s needs. It has been a statewide resource allocation as an alternative to the use of adaptive behavior scales	Three domains: daily living, health care, and behavioral	5-level scale (essentially independent to receive specialized assistance 24 h/day)	Internal consistency: ICC = 0.91 Test-retest: 0.82–0.93 Interrater: 0.84–0.88 Criterion validity: ICAP, DDP
PCANS [88]	Clinician reported questionnaire	5–17	Brain injury	Adapted from the CANS, it is a measure of support needs for children with brain injury, in terms of type and level of support need.	Four domains: special needs, basic activities of daily living, instrumental activities of daily living and informational and emotional supports	Section 1: items are endorsed if a support need is present. Section 2: from 0 = does not need contact to 7 = cannot be left alone	Test-retest: ICC = 0.98 Interrater: ICC = 0.93–0.96 Criterion validity with VABS, Wee-FIM, and KOSCHI
SIS [39,40,42,45,46,60,63,64,66,77,78,80,81,85,87,94,95,99,100,101]	Semi-structured interview to service users, their informal carers and staff	>16	Intellectual and developmental disabilities	It assesses the support needs that an adult with an intellectual disability needs to perform the activities of daily living	Home, community, employment, lifelong learning, health and safety, social activities, protection and advocacy, exceptional medical and behavioral support needs	Likert scale according to type of support (0–4, total physical support), frequency of support (0–4, always) and daily time (0–4, more than four hours per day)	Internal consistency: 0.44 to 0.91 Test-retest: 0.84–0.93 Interrater: 0.30–0.98 Criterion validity: ICAP, DDP, SNAP (see Appendix B)
SIS-C [31,32,33,37,52,53,56,76,77,78,82,83,96,97,104,105,106]	Semi-structured interview to informal carers and staff	5–16	Intellectual and developmental disabilities	It assesses the support needs that a child or adolescent with an intellectual disability needs to perform the activities of daily living	Home, community, school participation, school learning, health and safety, social activities, protection and advocacy, exceptional medical and behavioral support needs	Likert scale according to type of support (0–4, total physical support), frequency of support (0–4, always) and daily time (0–4, more than four hours per day)	Internal consistency: 0.83–0.94 Criterion validity: ARC-INICO Construct validity: 7 dimension and 3 correlated methods (see Appendix B)
SNAP [54,55]	Clinician reported questionnaire	>16	Diverse disabilities	It measures individual functional needs in areas of daily living. It produces a support profile, detailing the time allocations for staff support to assist in each area of need	Personal care, physical health, behavior support, night support, and social support	Scale from 1 (totally independent or no support required) to 5 (totally dependent on staff support)	Test-retest: 0.86–0.97 Interrater: ICC = 0.61–0.91 Concurrent validity with SIS, ICAP
SNQ [48]	Clinician reported questionnaire	24–76	Mental illness	It measures the support that people with severe mental illness need as a route to social inclusion	Community presence, community participation, choice and control, social roles and respect, skills and competencies, finance, and physical and mental health	Likert seven-point scales ranging from “No help” to “A great deal of help”. Physical and mental scales are scored by frequency on a seven-point scale (never to always)	Internal consistency: 0.48–0.84 Test-retest: 0.92 Criterion validity: MAC-2

^a^ CAN = Camberwell support needs; CANDID = Camberwell assessment of need for adults with developmental and intellectual disabilities; CANS = care and needs scale; I-CAN = instrument for classification and assessment of support needs; NC-SNAP = North Carolina—support needs assessment profile; PCANS = pediatric care and needs scale; SIS = supports intensity scale; SIS-C = supports intensity scale—children’s version; SNAP = service need assessment profile; SNQ = support needs questionnaire; ICC = internal consistency coefficient; GAF = global assessment of functioning scale; ICAP = inventory for client and agency planning; DDP = developmental disability profile; VABS = Vineland adaptive behavior scales.

## Appendix A. Search Strategy through Electronic Databases

**WEB OF SCIENCE. Databases = WOS Core Collection, Current Contents Connect, MEDLINE, SCIELO**	
#10	#4 AND #5 AND #9
#9	#6 OR #7 OR #8
#8	AK = ((((((disabilities OR disability) OR (disabled AND persons)) OR disabled) OR disablement) OR disablements) OR disabling)
#7	AB = ((((((disabilities OR disability) OR (disabled AND persons)) OR disabled) OR disablement) OR disablements) OR disabling)
#6	TI = ((((((disabilities OR disability) OR (disabled AND persons)) OR disabled) OR disablement) OR disablements) OR disabling)
#5	TI = (“support needs”) OR AB = (“support needs”) OR AK = (“support needs”)
#4	#1 OR #2 OR #3
#3	AK = (((((assess OR assessed) OR assessment) OR assesses) OR assessing) OR assessments) OR AK = (((((((((((evaluability OR evaluate) OR evaluated) OR evaluates) OR evaluating) OR evaluation) OR evaluations) OR evaluative) OR evaluatively) OR evaluatives) OR evaluator) OR evaluators) OR AK = (((((((((((((measurement OR measurements) OR measurability) OR measurable) OR measurably) OR measures) OR measureable) OR measured) OR measurer) OR measurers) OR measuring) OR measurings) OR measure) OR measures)
#2	AB = (((((assess OR assessed) OR assessment) OR assesses) OR assessing) OR assessments) OR AB = (((((((((((evaluability OR evaluate) OR evaluated) OR evaluates) OR evaluating) OR evaluation) OR evaluations) OR evaluative) OR evaluatively) OR evaluatives) OR evaluator) OR evaluators) OR AB = (((((((((((((measurement OR measurements) OR measurability) OR measurable) OR measurably) OR measures) OR measureable) OR measured) OR measurer) OR measurers) OR measuring) OR measurings) OR measure) OR measures)
#1	TI = (((((assess OR assessed) OR assessment) OR assesses) OR assessing) OR assessments) OR TI = (((((((((((evaluability OR evaluate) OR evaluated) OR evaluates) OR evaluating) OR evaluation) OR evaluations) OR evaluative) OR evaluatively) OR evaluatives) OR evaluator) OR evaluators) OR TI = (((((((((((((measurement OR measurements) OR measurability) OR measurable) OR measurably) OR measures) OR measureable) OR measured) OR measurer) OR measurers) OR measuring) OR measurings) OR measure) OR measures)
**Databases = APA PsycInfo CINAHL Complete; ERIC (through EBSCO interface)**	
#3	#1 OR #2
#2	AB (assess OR assessed OR assessment OR assesses OR assessing OR assessments OR evaluability OR evaluate OR evaluated OR evaluates OR evaluating OR evaluation OR evaluations OR evaluative OR evaluatively OR evaluatives OR evaluator OR evaluators OR measurement OR measurements OR measurability OR measurable OR measurably OR measures OR measureable OR measured OR measurer OR measurers OR measuring OR measurings OR measure OR measures) AND AB “support needs” AND AB (disabilities OR disability OR “disabled persons” OR disabled OR disablement OR disablements OR disabling OR AB disabilities OR disability OR “disabled persons” OR disabled OR disablement OR disablements OR disabling)
#1	TI (assess OR assessed OR assessment OR assesses OR assessing OR assessments OR evaluability OR evaluate OR evaluated OR evaluates OR evaluating OR evaluation OR evaluations OR evaluative OR evaluatively OR evaluatives OR evaluator OR evaluators OR measurement OR measurements OR measurability OR measurable OR measurably OR measures OR measureable OR measured OR measurer OR measurers OR measuring OR measurings OR measure OR measures) AND TI “support needs” AND TI (disabilities OR disability OR “disabled persons” OR disabled OR disablement OR disablements OR disabling OR AB disabilities OR disability OR “disabled persons” OR disabled OR disablement OR disablements OR disabling)
**Database = PUBMED**	
#4	#1 AND #2 AND #3
#3	(“disabilities”[Title/Abstract] OR “disability”[Title/Abstract] OR “disabled persons”[MeSH Terms] OR (“disabled”[Title/Abstract] AND “persons”[Title/Abstract]) OR “disabled persons”[Title/Abstract] OR “disabled”[Title/Abstract] OR “disablement”[Title/Abstract] OR “disablements”[Title/Abstract] OR “disabling”[Title/Abstract])
#2	“support needs”[Title/Abstract]
#1	(“assess”[Title/Abstract] OR “assessed”[Title/Abstract] OR “assessment”[Title/Abstract] OR “assesses”[Title/Abstract] OR “assessing”[Title/Abstract] OR “assessments”[Title/Abstract] OR “evaluability”[Title/Abstract] OR “evaluate”[Title/Abstract] OR “evaluated”[Title/Abstract] OR “evaluates”[Title/Abstract] OR “evaluating”[Title/Abstract] OR “evaluation”[Title/Abstract] OR “evaluations”[Title/Abstract] OR “evaluative”[Title/Abstract] OR “evaluatively”[Title/Abstract] OR “evaluatives”[Title/Abstract] OR “evaluator”[Title/Abstract] OR “evaluators”[Title/Abstract] OR “measurement”[Title/Abstract] OR “measurements”[Title/Abstract] OR “measurability”[Title/Abstract] OR “measurable”[Title/Abstract] OR “measurably”[Title/Abstract] OR “measures”[Title/Abstract] OR “measureable”[Title/Abstract] OR “measured”[Title/Abstract] OR “measurer”[Title/Abstract] OR “measurers”[Title/Abstract] OR “measuring”[Title/Abstract] OR “measurings”[Title/Abstract] OR “measure”[Title/Abstract] OR “measures”[Title/Abstract])
**Database = SCOPUS**	
#1	(TITLE-ABS-KEY (assess OR assessed OR assessment OR assesses OR assessing OR assessments OR evaluability OR evaluate OR evaluated OR evaluates OR evaluating OR evaluation OR evaluations OR evaluative OR evaluatively OR evaluatives OR evaluator OR evaluators OR measurement OR measurements OR measurability OR measurable OR measurably OR measures OR measureable OR measured OR measurer OR measurers OR measuring OR measurings OR measure OR measures) AND TITLE-ABS-KEY (“support needs”) AND TITLE-ABS-KEY (disabilities OR disability OR “disabled persons” OR disabled OR disablement OR disablements OR disabling))
**Database = PROQUEST Central**	
#9	#4 OR #8
#8	#5 AND #6 AND #7
#7	ab(disabilities OR disability OR “disabled persons” OR disabled OR disablement OR disablements OR disabling OR AB disabilities OR disability OR “disabled persons” OR disabled OR disablement OR disablements OR disabling))
#6	ab(“support needs”)
#5	ab(assess OR assessed OR assessment OR assesses OR assessing OR assessments OR evaluability OR evaluate OR evaluated OR evaluates OR evaluating OR evaluation OR evaluations OR evaluative OR evaluatively OR evaluatives OR evaluator OR evaluators OR measurement OR measurements OR measurability OR measurable OR measurably OR measures OR measureable OR measured OR measurer OR measurers OR measuring OR measurings OR measure OR measures)
#4	#1 AND #2 AND #3
#3	ti(disabilities OR disability OR “disabled persons” OR disabled OR disablement OR disablements OR disabling OR AB disabilities OR disability OR “disabled persons” OR disabled OR disablement OR disablements OR disabling))
#2	ti(“support needs”)
#1	ti(assess OR assessed OR assessment OR assesses OR assessing OR assessments OR evaluability OR evaluate OR evaluated OR evaluates OR evaluating OR evaluation OR evaluations OR evaluative OR evaluatively OR evaluatives OR evaluator OR evaluators OR measurement OR measurements OR measurability OR measurable OR measurably OR measures OR measureable OR measured OR measurer OR measurers OR measuring OR measurings OR measure OR measures)
**Database = CSIC**	
#4	#1 AND #2 AND #3
#3	[Por Campos] Título documento(Todas las palabras) = “discapacidad “ O Resumen documento(Todas las palabras) = “discapacidad “ O Palabras clave Autor(Todas las palabras) = “discapacidad”
#2	[Por Campos] Título documento(La frase) = ““necesidades de apoyo”“ O Resumen documento(La frase) = “necesidades de apoyo”“ O Palabras clave Autor(La frase) = ““necesidades de apoyo”
#1	[Por Campos] Título documento(Alguna palabra) = “evaluación evaluar evaluador medir medida” O Resumen documento(Alguna palabra) = “evaluación evaluar evaluador medir medida” O Palabras clave Autor(Alguna palabra) = “evaluación evaluar evaluador medir medida”

## Appendix B. Validity and Reliability of the Supports Intensity Scales

**Table A1:** Association Indices Between Areas of Support Needs.

Study	Cronbach’s Alpha	Correlation Coefficients between			SIS
		Subscales	Applications	Evaluators	
Adam-Alcocer & Giné (2013)	0.83–0.94 (a–g); 0.91 (total)	0.28–0.84 (a–g)	-	-	C
Arnkelsson & Sigurdsson (2014)	0.78–0.90 (a–f); 0.97 (total)	-	-	-	A
Arnkelsson & Sigurdsson (2016)	0.9–0.95 (a–f); 0.98 (total)	0.60–0.80 (a-f)	-	-	A
Brown et al. (2009)	-	0.72–0.88 (a–f)	-	-	A
Chou et al. (2013)	0.87–0.93 (a–f); 0.96 (total)	0.90–0.93 (a–f)	-	-	A
Claes et al. (2009)	-	0.48–0.88 (a–f)	-	0.30–0.77	A
Cruz et al. (2010)	0.95–0.99 (a–f); 0.99 (total)	-	-	-	A
Guillén et al. (2015)	0.95–0.97 (a–g); 0.99 (total)	-	-	-	C
Guillén et al. (2017)	0.95–0.97 (a–g); 0.99 (total)	-	-	-	C
Jenaro et al. (2011)	0.83–0.94 (a–f); 0.97 (total)	0.61–0.82 (a–f)	-	0.67–0.98	A
Lamoureux-Hébert & Morin (2009)	0.89–0.95 (a–f); 0.98 (total)	-	-	-	A
Morin & Cobigo (2009)	-	-	-	0.79–0.92	A
Shogren et al. (2014)	0.90–0.97 (a–f); 0.98 (total)	0.79–0.90 (a–f)	-	0.71	A
Smit et al. (2011)	0.44–0.82 (a–f)	0.44–0.82 (a–f)	-	-	A
Thompson et al. (2002)	0.97–0.98 (a–f)	0.45–0.87 (a–f)	-	-	A
Thompson et al. (2008)	-	-	-	0.36–0.93	A
Thompson et al. (2014)	0.93–0.95 (a–g)	0.61–0.85 (a–g)	-	-	C
Vega Córdoba et al. (2014)	0.93–0.97 (a–f)	0.77–0.89 (a–f)	-	-	A
Verdugo et al. (2010)	0.90–0.99 (a–f)	0.78–0.88 (a–f)	0.84–0.93	0.60–0.84	A

Note. SIS = supports intensity scale; A = adult version; C = children version; (a–f) = SIS subscales including: a (home activities), b (community), c (lifelong learning), d (employment), e (health and safety) and f (social activities); (a–g) = SIS-C subscales including: a (home activities), b (community), c (school participation), d (school learning), e (health and safety), f (social activities) and g (advocacy); (total) = overall index of support needs.

**Table A2:** Fit Indices of the Support Needs Measurement Factorial Models.

Study ^a^	χ^2^ (df)	RMSEA (CI)	CFI	TLI	SRMR	SIS
**One-dimensional model**						
Bossaert et al. (2009)	34,560.72 (1127)	0.160	0.920		0.095	A
Kuppens et al. (2010)	27,550.33 (2224)	0.061	0.990	-	0.051	A
Verdugo, Amor, et al. (2019)	9382 (189)	0.217 (0.210–0.220)	0.705	0.672	-	C
Verdugo, Guillén, et al. (2016)	4625.11 (189)	0.170 (0.170–0.170)	0.960	0.950	0.047	C
**Oblique model**						
Aguayo, Arias, et al. (2019)	1587 (168)	0.201 (0.19–0.21)	0.681	0.600	0.134	C
Bossaert et al. (2009)	11,767.19 (1112)	0.091	0.975	-	0.079	A
Guillén et al. (2017)	1200.35 (168)	0.080 (0.076–0.085)	0.990	0.990	0.028	C
Kuppens et al. (2010)	8628.27 (1112)	0.033	0.997	-	0.032	A
Seo, Shogren, et al. (2017)	9565.91 (596)	0.066 (0.064–0.067)	0.969	0.960	-	A
Seo, Wehmeyer, et al. (2017)	2969.12 (1003)	0.109	0.895	0.884	-	A
Shogren et al. (2015)	4547.77 (1008)	0.072 (0.070–0.075)	0.968	0.960	-	C
Shogren et al. (2016)	75,919.30 (149)	0.061 (0.061–0.062)	0.979	0.973	0.021	A
Shogren et al. (2018)	182.76 (69)	0.142 (0.117–0.167)	0.887	0.851	-	A
Verdugo, Amor, et al. (2019)	7623.00 (168)	0.207 (0.200–0.210)	0.761	0.701	-	C
Verdugo, Arias, et al. (2016)	6676.80 (2016)	0.079 (0.077–0.081)	0.964	0.955	-	C
Verdugo, Guillén, et al. (2016)	981.57 (168)	0.077 (0.073–0.082)	0.990	0.990	0.020	C
**Second-order model**						
Verdugo, Arias, et al. (2019)	6599.40 (168)	0.217	0.722	0.652	-	C
Verdugo, Guillén, et al. (2016)	1402.92 (182)	0.091 (0.086–0.095)	0.990	0.990	0.033	C
**Correlated traits—orthogonal methods model**						
Aguayo, Arias, et al. (2019)	334 (147)	0.078 (0.06–0.08)	0.958	0.940	0.034	C
Verdugo, Arias, et al. (2019)	587.5 (147)	0.059	0.982	0.974	-	C
**Correlated traits—correlated methods model**						
Aguayo, Arias, et al. (2019)	330 (144)	0.078 (0.06–0.08)	0.958	0.939	0.026	C
Verdugo, Amor, et al. (2019)	633 (144)	0.057 (0.050–0.060)	0.984	0.977	-	C
Verdugo, Arias, et al. (2019)	554.3 (144)	0.059	0.982	0.974	-	C

Note. SIS = supports intensity scale; A = adult version; C = children version; χ^2^ (df) = Chi-square (degrees of freedom), with χ^2^ (*p* < α); RMSEA (CI) = root-mean square error of approximation (confidence interval), with values 0.05 to 0.08 (good fit); CFI = comparative fit index, with values 0 (no fit) to 1 (>0.90, best fit); TLI = Tucker–Lewis fit index, with values 0 (no fit) to 1 (>0.90, best fit); SRMR = Standardized root-mean square residual, with values <0.05 (good fit). In the cases of invariance measurement studies that do not provide structure fit data, the fit indices of the configural model have been included. ^a^ The explored factorial models are the following: (a) one-dimensional model, where the support needs are explained by a single general factor; (b) oblique model, where the support needs are explained by correlated factors relating to the SIS’ support areas; (c) second-order model, where the support needs are explained by a general factor influencing the different SIS’ support areas; (d) correlated traits—orthogonal methods model, where the support needs are explained by correlated factors relating to the SIS’ support areas and three method factors (type, frequency, and daily support time); and (e) correlated traits—correlated methods model, where the support needs are explained by correlated factors relating to the SIS’ support areas and three correlated method factors (type, frequency, and daily support time).
